# Hierarchical Deep Learning Framework for Skin Disease and Cancer Classification Performance Enhancement

**DOI:** 10.3390/s26092833

**Published:** 2026-05-01

**Authors:** Chanapa Chaitan, Sasithorn Tengjongdee, Suejit Pechprasarn, Kitsada Thadson

**Affiliations:** 1 College of Biomedical Engineering, Rangsit University, Pathum Thani 12000, Thailand; chanapa.c64@rsu.ac.th (C.C.); sasithorn.t65@rsu.ac.th (S.T.); suejit.p@rsu.ac.th (S.P.); 2 Center of Excellence in Artificial Intelligence & Supercomputing, Rangsit University, Pathum Thani 12000, Thailand

**Keywords:** multi-class classification, hierarchical binary classification, skin cancer, deep learning, convolutional neural networks

## Abstract

Currently, the number of people who have been investigated for skin cancer has increased significantly worldwide. For prior diagnosis, dermatologists can typically visually inspect skin lesions for abnormalities. However, an expert is required, and the similarity of some skin lesions remains challenging. This study aimed to address the challenge of classifying multiple images of skin conditions, including both Benign and Malignant groups, using the hierarchical method. Instead of directly performing multi-class classification using a single model, multiple binary classification models were organized to reduce task complexity and improve overall performance. In the methodology, four convolutional neural network (CNN) models, namely MobileNetV2, EfficientNet-B0, ResNet-18, and ResNet-50, were selected as candidates for this problem. The proposed hierarchical binary classification model was evaluated against conventional multi-class classification methods. As a result, various evaluation metrics were used to assess model performance, with recall as the primary metric in this study, given the emphasis on minimizing false negatives. However, some results revealed discrepancies between the highest recall and other performance metrics. Further analysis demonstrated the potential of using recall as a selection criterion for identifying the most suitable CNN models. The single model-based classification of six classes of skin lesion images achieves the highest recall of 60.27% with MobileNetV2. Meanwhile, the proposed hierarchical model achieves a higher recall of 82.62%, representing a significant increase of 22.35%. Additionally, improvements were observed across all other evaluation metrics, including accuracy (+25.46%), precision (+17.21%), F1-score (+21.34%), balanced accuracy (+12.69%), specificity (+3.03%), and G-mean (+14.25%). These improvements indicate enhanced performance in correctly identifying both positive and negative cases, while reducing misclassification rates. This outcome demonstrates the potential to improve the model’s generalizability, thereby increasing its applicability across various clinical decision-support systems.

## 1. Introduction

Epidemiological studies have shown a consistent global rise in skin cancer cases over recent decades [1,2]. By 2024, the number of new cases worldwide is expected to exceed 200,000 [3]. Although Thailand has a lower incidence rate compared to other tropical countries, the trend continues to rise year after year [4]. Dermatological conditions represent a clinically diverse spectrum of diseases [5], ranging from benign disorders such as seborrheic keratosis to lesions with malignant potential, including nevus and actinic keratosis. Changes in skin conditions, such as chronic wounds, and noticeable alterations in the size, color, or shape of a mole can also increase the risk. Certain conditions can progress to various forms of skin cancer [6], most commonly basal cell carcinoma, squamous cell carcinoma, and melanoma. Among these, melanoma is the most aggressive type with a high potential for metastasis.

Furthermore, the accurate diagnosis of the six dermatological conditions: Basal Cell Carcinoma, Squamous Cell Carcinoma, Melanoma, Seborrheic Keratosis, Actinic Keratosis, and Nevus. These are of paramount medical importance. This spectrum includes both malignant lesions and benign conditions, which causes a significant challenge for accurate visual differential diagnosis [7]. Diagnosing dermatological conditions, especially during the early stages of skin cancers such as melanoma, is exceptionally challenging. This is because clinical manifestations are often subtle and nonspecific in the initial phases, making it difficult to distinguish clearly between malignant and benign lesions [8,9]. Current diagnostic methods, which rely on visual inspection and dermoscopy, are heavily dependent on dermatologists’ skill and experience [10], resulting in several significant limitations in clinical settings. Furthermore, accessibility issues present a critical constraint. The shortage of dermatology specialists in underserved and rural areas exacerbates the problem of delayed access to quality diagnosis [11]. To address these challenges, hierarchical binary classification models have emerged as a promising approach for simplifying complex tasks in medical image analysis.

Nowadays, various artificial intelligence (AI) techniques have become integral to advancements in biomedical and medical fields [10]. With the potential of state-of-the-art machine learning algorithms, particularly deep learning models, applications in these fields have continued to evolve in several domains, including biomedical signal processing [12,13], biomedical sensing and imaging [14,15], and medical image analysis [16,17]. Additionally, among several types of techniques, convolutional neural networks (CNNs) have gained widespread recognition for their exceptional performance in analyzing medical imaging data [18]. CNNs are particularly well-suited for analyzing grid-like structured data, such as medical images [19], making them highly adaptable and effective for a wide range of clinical applications. They leverage their ability to perform automatic feature extraction from images [20], for example, diagnosing skin cancer [21], identifying diseases from chest X-rays [22], locating tumors in mammograms [8], as well as precisely delineating the boundaries of tumors in MRI scans [23].

For dermatological diagnosis, several CNN models [24,25] demonstrate strong performance for distinguishing between malignant and benign lesion groups in skin lesion images. However, most existing approaches rely on single-model-based multiclass classification frameworks, which face dramatic challenges in real-world applications. These include class imbalance [26], increasing model complexity with a high number of classes [27], and similarity between different classes [28]. Although techniques such as undersampling, oversampling, and classifier-level optimization have been proposed to mitigate these issues, they often provide only limited improvements. They may lead to information loss or overfitting. Moreover, such approaches do not fundamentally address the difficulty of learning discriminative features across multiple similar classes within a single unified model. As a result, there remains a need for more effective strategies that can simplify the classification task while improving robustness and accuracy.

Hierarchical binary classification is an integrated architecture that combines multiple classification models [29]. This assembled framework can overcome multi-classification challenges by reducing model complexity by adjusting learnable parameters for each class pair separately, rather than adapting to every investigated class, and then merging them into a tree structure. The potential of this technique, moreover, provides performance improvement in terms of loss and accuracy for both training and testing [30].

In recent years, advanced strategies have been demonstrated to enhance image classification performance. By combining multiple CNN architectures, ensemble methods achieve their success in addressing the generalization limitations in medical image classification and achieve improved accuracy compared to individual models [17,31]. Moreover, hierarchical classification techniques can address multiclass complexity by separating tasks into subtasks for binary decisions, which enhances the model’s capability to learn for an imbalanced dataset and different class similarity more effectively [32]. However, the application of these frameworks to dermatological image classification remains limited, especially on sophisticated datasets.

To address these challenges, this study proposes a novel multiclassification approach based on a hierarchical binary classification architecture integrated with CNNs for skin cancer image classification, using two distinct public datasets. Unlike conventional multiclass classification methods that use a single model, this proposed technique constructs a model from multiple binary classifiers. In addition, this work systematically compares multiple well-known CNN architectures within both conventional and proposed hierarchical frameworks to evaluate their performance on a sophisticated dataset. The proposed approach aims to enhance classification performance and model generality, particularly in imbalanced datasets and ambiguous class scenarios.

## 2. Materials and Methods

### 2.1. Dataset

The dataset comprises images of dermatological abnormalities from two public sources, including PAD-UFES-20 and HAM10000 [33,34].

The PAD-UFES-20 dataset includes images of six distinct conditions: Basal Cell Carcinoma (BCC), Squamous Cell Carcinoma (SCC), Melanoma (MEL), Actinic Keratosis (ACK), Seborrheic Keratosis (SEK), and Nevus (NEV). It contains 2298 images from 1373 patients. The image dimensions range from 187 × 187 × 3 pixels to 3024 × 3024 × 3 pixels, and all files are in PNG format. As mentioned, the PAD-UFES-20 dataset is relatively small. Therefore, the HAM10000 dataset was incorporated to address this limitation.

The HAM10000 dataset is a publicly available collection comprising 10,015 dermatological images of BCC, MEL, ACK, SEK, and NEV. The total image dimensions are 600 × 450 × 3 pixels in JPG format.

For data collection, the PAD-UFES-20 dataset was collected on a smartphone in a typical uncontrolled environment; in contrast, the HAM10000 dataset was collected with a dermoscope in a controlled environment. This work aims to improve the generalizability of the classification model by combining these two datasets to expand its applicability to both low and high levels of environmental control.

Both the PAD-UFES-20 and HAM10000 datasets are publicly available resources distributed under open licenses for non-profit academic research. The original publishers have anonymized all images to ensure compliance with ethical guidelines and standards. Therefore, the use of these datasets in this study is appropriate and fully complies with the terms of use.

### 2.2. Data Preparation

To standardize input to the neural network and enhance training efficiency, dermatological images of varying sizes were resized to a uniform 224 × 224 × 3 pixels to match the input layer size of the selected CNN models. As the data were derived from two different datasets with imbalanced original class distributions, a balancing strategy was applied. Each class was expanded to 3500 images using structured augmentation, which is approximately half of the maximum number of images in the NEV class after dataset combination and splitting.

**Figure 1:**
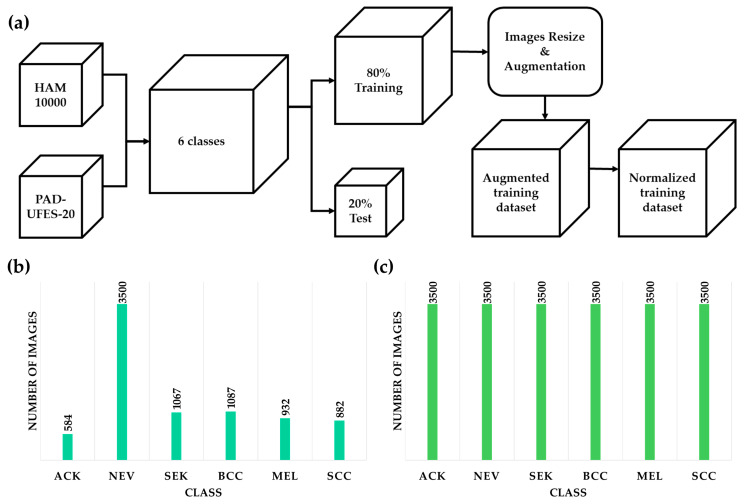
(**a**) Data preparation process from combining two datasets to dataset balancing using augmentation methods. (**b**) classes distribution in the training dataset before augmentation and (**c**) after augmentation.

Firstly, the images will be randomly scaled between 80% and 110% of their original size. Secondly, the random-image-reflection process is applied to the synthesized data. The empty areas generated during those transformations will be filled with zeros to maintain the required image size. The original training dataset was repeatedly augmented until the desired number of images per class was achieved, resulting in each training directory containing exactly 3500 images per category, as shown in Figure 1. Meanwhile, 20% of the original image dataset will be randomly reserved for model testing without augmentation to reflect real-world scenarios better. Additionally, to prevent information leakage that could lead to inaccurate evaluation, both the training and testing datasets were manually inspected at the individual-image level.

### 2.3. Convolutional Neural Networks Models

The four novel CNN models are selected to experiment with different skin diseases as image classification classes. Additionally, these models were investigated for both individual multi-class classification and hierarchical binary classification methods.

MobileNetV2 [35] is a CNN architecture specifically designed for mobile and embedded vision applications. It introduces an inverted residual structure with linear bottlenecks to improve representational power while minimizing computational complexity. With a total of approximately 3.4 million trainable parameters and 53 layers. Owing to its compact design and high processing efficiency, this model has been extensively applied in various domains, including image classification [36], particularly on mobile [37] and edge computing devices [38].

EfficientNet-B0 [39] employs a compound scaling strategy that uniformly scales the network’s depth, width, and resolution to achieve an optimal balance between accuracy and computational efficiency. With approximately 5.3 million trainable parameters and 237 layers, this architecture enables high-performance image analysis while maintaining a compact model size. Owing to its lightweight and versatile design, the model has been widely applied in various computer vision tasks, including image classification [40], image segmentation [41], and transfer learning [42].

ResNet-18 [8] employs a residual learning framework to address the vanishing gradient problem in deep networks. It uses the identity-mapping principle via shortcut connections, enabling the network to learn only the residual function rather than the entire underlying mapping. With its shallow 18-layer architecture and 11.7 million trainable parameters, ResNet-18 enables fast image analysis and feature extraction. Generally, this residual model has been widely applied in various fields, such as image classification [40]. Object Detection [43] and Feature Extraction [44].

ResNet-50 [8] employs the same residual learning framework as ResNet-18 but incorporates a deeper architecture to enhance feature representation and model accuracy. It utilizes bottleneck residual blocks that reduce the number of parameters while maintaining efficient information flow through identity shortcut connections. The model comprises approximately 25.6 million trainable parameters and 50 layers. ResNet-50 achieves high computational efficiency while extracting complex image features. Due to its robustness and precision, this model has been extensively applied in diverse fields, including image classification [45] and advanced computer vision tasks [46].

To summarize, the four models studied differ in several domains, including model structure, loss function, and the number of layers. However, they all demonstrated significant potential for image classification, as evidenced by the existing literature. For this reason, they are selected to participate in this investigation of hierarchical binary architecture performance.

### 2.4. Classification Strategy

#### 2.4.1. Multi-Class Classification

**Figure 2:**
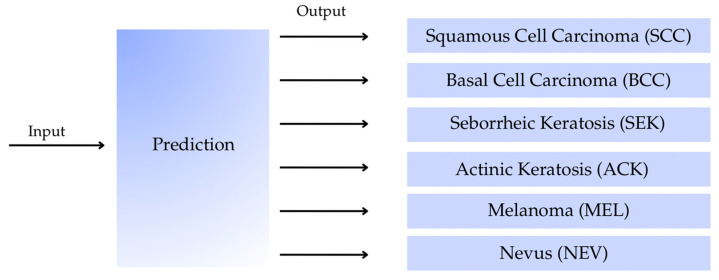
The diagram illustrates multi-class classification models using CNNs to predict six classes of skin lesions from dermatological images.

Traditionally, the multi-class classification targets are performed using a single model. Additionally, the model can be optimized using various techniques to achieve the lowest loss or the highest classification accuracy. This conventional strategy will be used to evaluate the classification model for identifying six classes of dermatological lesion images, as shown in Figure 2, using several well-known CNN architectures.

#### 2.4.2. Hierarchical Classification

**Figure 3:**
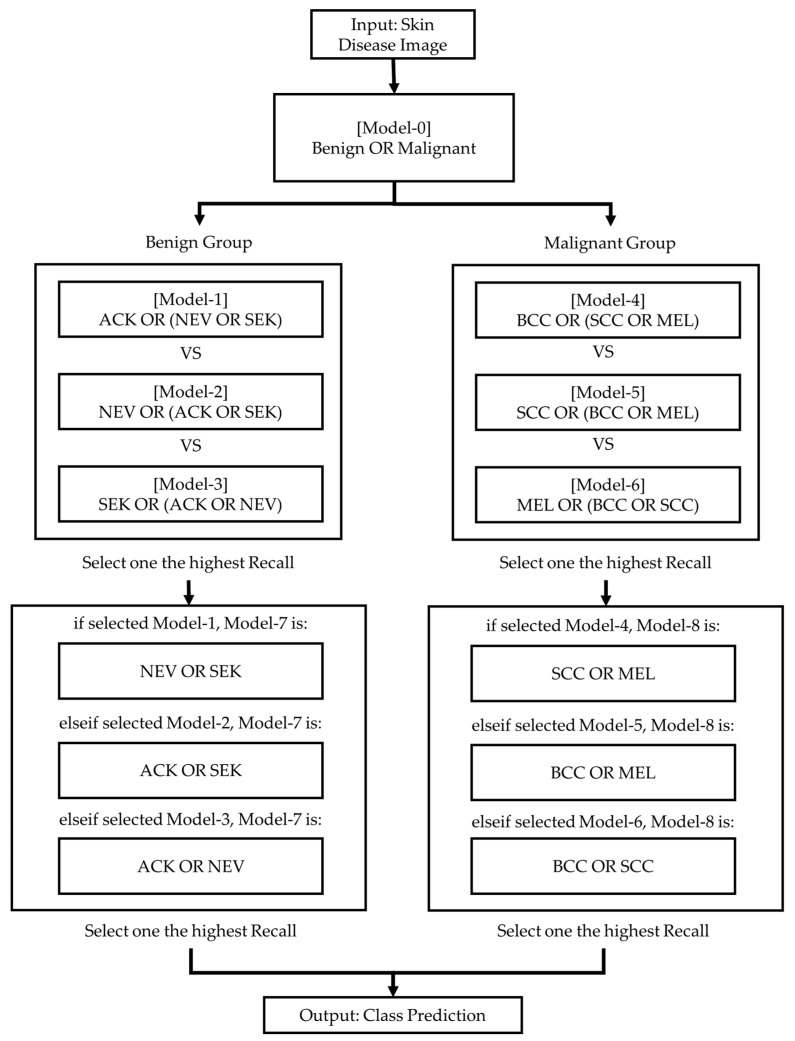
The flowchart illustrates the Hierarchical classification strategy for the six types of dermatological lesions.

A hierarchical classification model that combines multiple predictive models was proposed in this work. Given the models’ unique potential, this combination could improve overall performance. In addition, the structured model has the advantages of being separately and adaptively modified by splitting the main task into several subtasks. In this work, the hierarchical classification model was structured from different binary classification models, as shown in Figure 3.

Initially, the input images are classified into two groups—benign and malignant—using the investigated CNN models. Subsequently, within the benign group, pairwise combinations among the three classes are evaluated to identify the optimal models based on the recall metric. Similarly, the three classes within the malignant group are processed identically. In the next phase, the remaining two classes in each group are further examined. Finally, a hierarchical classification structure is constructed based on the most suitable models for subsequent performance evaluation.

### 2.5. Training Procedure

After data preparation, the transfer learning technique was employed to fine-tune the well-pretrained model parameters on our dataset. Those four selected CNN models were trained based on the same training configuration, including the adaptive momentum estimation (Adam) optimizer [47], batch size of 20, constant learning rate of 0.001, and total training for 50 epochs to ensure the optimal loss convergence.

As shown in Table 1, the model was trained using the Adam optimizer with standard parameter settings, including a gradient decay factor of 0.9, a squared gradient decay factor of 0.999, and an epsilon of 1 × 10^−8^ to ensure numerical stability. The initial learning rate was fixed at 1 × 10^−3^ with no learning rate scheduling applied throughout training. The model was trained for a maximum of 300 epochs using a mini-batch size of 40, and the training data were shuffled at every epoch to maintain stochasticity. Validation was performed periodically using an augmented image datastore with a frequency of 10 iterations, with no early stopping mechanism (infinite validation patience). Model checkpoints were saved at every epoch to monitor training progress.

For regularization, L2 regularization was applied with a coefficient of 1 × 10^−4^ to constrain the magnitude of the model weights. Additional implicit regularization was introduced through data augmentation in the validation pipeline and through Batch Normalization with automatically computed statistics. The dataset was shuffled at each epoch to reduce overfitting due to data ordering. Gradient clipping was defined using the L2 norm method.

According to the classification strategy, the models are trained for both multi-classification (six classes) and binary classification (two classes). The latter are investigated individually before being assembled into an optimal hierarchical classification structure for six classes. Lastly, the total number of training models is 4 for multi-classification and 20 for the investigated binary classification models.

The computing resources for training, testing, and evaluating the model’s performance are operated on various hardware as shown in Table 2. Additionally, this work was developed in MATLAB 2024A.

### 2.6. Model Evaluation and Measurement

To determine the most effective deep learning model for skin disease classification, 4 models were tested. All models were evaluated using the same performance matrix, which included Accuracy, Precision, Recall, F1-score, Specificity, Balanced Accuracy, and G-mean.

Accuracy is one of the primary metrics used to evaluate a deep learning model’s performance. It measures the proportion of all correct predictions relative to the total number of data points, which can be calculated using Equation (1):

(1) $$Accuracy=\frac{TP+TN}{TP+TN+FP+FN}$$

where

True positive (TP) is the number of correct predictions for the class as a positive assumption.

True negative (TN) is the number of correct predictions for the class as a negative assumption.

A false positive (FP) is the number of incorrect predictions for the class when the assumption is that the class is positive.

False negative (FN) is the number of incorrect predictions for the class as a negative assumption.

Precision is a metric that indicates the model’s reliability in making optimistic predictions. It focuses on the proportion of true positive predictions among all instances that the model predicted as positive. It is calculated using Equation (2):

(2) $$Precision=\frac{TP}{TP+FP}$$

Recall measures the model’s ability to predict each class correctly. It focuses specifically on TP by considering the proportion of correctly identified positive instances out of all actual positive instances, which can be calculated using Equation (3):

(3) $$Recall=\frac{TP}{TP+FN}$$

The F1-score is particularly useful when you need to find a balance between Precision and Recall, especially in situations where the class distribution is imbalanced. It provides a single metric that considers both FP and FN. F1-Score is the mean of the prediction accuracy of Precision and Recall, which can be calculated as described in Equation (4):

(4) $$F1-\mathrm{Score}\;=\frac{2\times (Precision\times Recall)}{Precision+Recall}$$

Specificity is essential for assessing how well a model correctly identifies negative cases. This value is particularly useful when a false positive prediction has serious consequences. Moreover, specificity is also known as the True Negative Rate (TNR), which measures how well the model correctly predicts the absence of a condition and avoids false alarms. It can be calculated using Equation (5):

(5) $$Specificity=\frac{TN}{TN+FP}$$

Balanced Accuracy is used for both binary and multi-class classification. This value is the arithmetic mean of Recall and Specificity. It is often employed when dealing with imbalanced data. This refers to situations where one class in the data appears much more frequently than another class, and it can be calculated using Equation (6):

(6) $$Balanced\;Accuracy=\frac{Recall+Specificity}{2}$$

The G-Mean is the geometric mean of Sensitivity and Specificity. It has the advantage of considering ratios and rates. Therefore, using the G-Mean as a metric for classification problems is more appropriate, especially for this study, and it can be calculated using Equation (7):

(7) $$G-\mathrm{Mean}\;=\sqrt{Recall\times Specificity}$$

To evaluate results accurately and measure the model’s reliability, the training accuracy will be calculated as an average across repeated runs, and the average across classes (macro-average) will be reported for each model for comparison.

Additionally, given the class imbalance between benign and malignant samples, evaluation was primarily based on Recall. Recall was prioritized because correctly identifying malignant cases is clinically critical. In medical diagnosis, a false negative failing to detect a malignant sample carries far greater risk than a false positive. Recall directly measures the model’s ability to capture all actual malignant cases, thereby helping ensure that harmful conditions are not overlooked. For these reasons, the recall metric will be utilized to select the optimal model for constructing the hierarchical structure, prioritizing the highest recall values. In cases where two or more models achieve identical recall values, F1-score, precision, and accuracy will be considered sequentially.

### 2.7. Gradient Camera (Grad-CAM)

In the hierarchical classification of skin diseases, the process of Gradient Camera (Grad-CAM) visualizations to identify skin disease pathology begins with calculating the importance weights ($\alpha_{k}^{c}$) as shown in Equation (8). This is achieved by computing the gradient of the prediction score for a specific disease class with respect to the feature maps of the final convolutional layer, followed by Global Average Pooling to determine how much the morphological characteristics in those maps influence the model’s decision.

(8) $$\alpha_{k}^{c}=\frac{1}{Z}\sum_{i}\;\sum_{j}\frac{\partial y^{c}}{\partial A_{ij}^{k}}\;$$

where

$\alpha_{k}^{c}$: Importance weight of feature map *k* for class *c*.
$y^{c}$: Prediction score for class *c* (before Softmax).
$A_{ij}^{k}$: Pixel value at position (*i*, *j*) in feature map *k*.
***Z***: Total number of pixels in the feature map (used for averaging).
$\frac{\partial y^{c}}{\partial A_{ij}^{k}}$: The gradient represents the influence of the pixel on the prediction.

Grad-CAM Heatmap $\left(L_{Grad-CAM}^{c}\right)$ is generated according to Equation (9) by performing a weighted sum of the feature maps and applying the ReLU function to filter only the significant positive features.

(9) $$L_{Grad-CAM}^{c}=ReLU\left(\sum_{k}\alpha_{k}^{c}A^{k}\;\right)$$

where

$L_{Grad-CAM}^{c}$: the resulting heatmap displayed in various colors.
$\sum_{k}\alpha_{k}^{c}A^{k}$: Weighted sum of all feature maps.
ReLU: Function that keeps only positive values (highlighting disease pathology).

When these numerical values are processed, they are translated into different colors: Red represents the highest values, indicating critical indicators of the lesion’s pathology; Yellow represents areas of secondary importance; and Blue represents low or zero values [48], indicating normal skin tissue that the model did not find significant for its prediction.

## 3. Results

Four deep learning models were trained and tested: MobileNetV2, EfficientNet-B0, ResNet-18, and ResNet-50. The results presented in this section represent the optimal performance of each model group. The data includes detailed numerical results, including Confusion Matrices, which serve as the basis for calculating and evaluating model performance using various evaluation metrics.

### 3.1. Models Performances

#### 3.1.1. Multi-Class Classification

**Figure 4:**
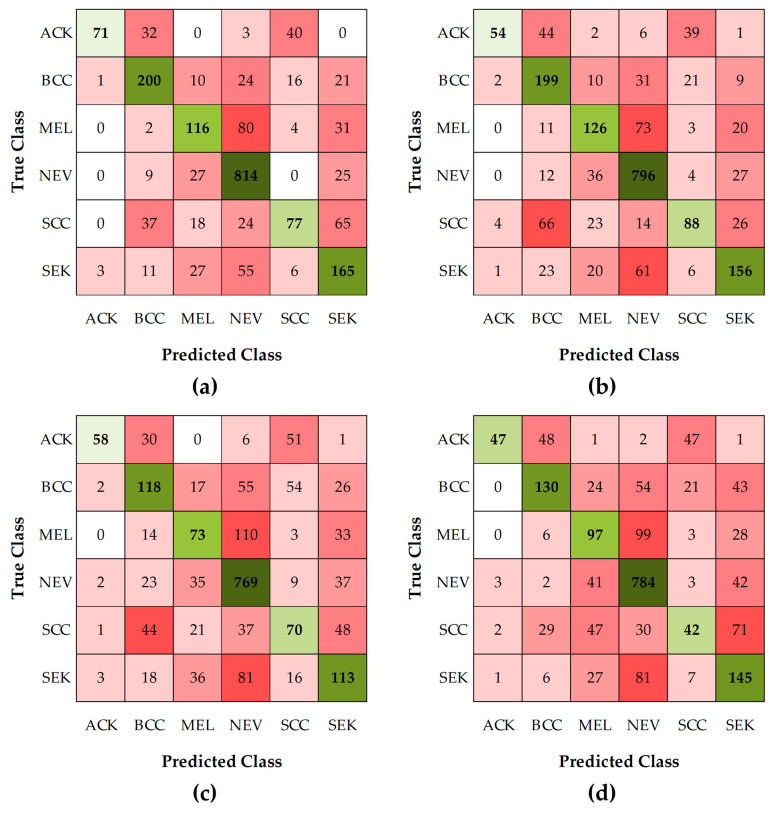
This is a figure displaying the Multi-Class Classification Results Across All Six Types of Dermatological Lesions as: (**a**) Confusion Matrix for the MobileNetV2 Model; (**b**) Confusion Matrix for the EfficientNetB0 Model; (**c**) Confusion Matrix for the ResNet-18 Model; (**d**) Confusion Matrix for the ResNet-50 Model.

Multi-Class Skin Lesion Classification. The accuracy performance of the 4 models in this group ranged from 40% to 60%, as shown in Table 3. The best predictive model was MobileNetV2, which achieved a recall of 60.27%, as shown in Figure 4. However, this result also suggests that the model is not sufficiently powerful to classify lesions reliably.

#### 3.1.2. Hierarchical Binary Classification

The results of the evaluation of the binary skin lesion classification model were divided into 5 groups: Benign and Malignant group, Benign group with the best prediction performance, Malignant group with the best prediction performance, and Malignant group.

Classification of skin lesions into Benign and Malignant groups, into Benign and Malignant only groups. Table 4 presents the performance of the 4 models with evaluation indicators Accuracy, Precision, Recall, F1-score, Balanced Accuracy, Specificity, and G-Mean.

Classification of skin lesions in the Benign group. This group is divided into 3 subgroups: ACK and NEV + SEK group, NEV and ACK + SEK group, SEK and ACK + NEV group, as shown in Table 5, with evaluation indicators Accuracy, Precision, Recall, F1-score, Balanced Accuracy, Specificity, and G-Mean.

Classification of skin lesions in the Benign group. This group is divided into 3 subgroups: ACK and NEV + SEK, NEV and ACK + SEK, and SEK and ACK + NEV. Of these 3 groups, the highest-performing group is the ACK and NEV + SEK group. In this group, the results are from NEV and SEK. Table 6 presents the performance of the 4 models, evaluated using the following indicators: Accuracy, Precision, Recall, F1-score, Balanced Accuracy, Specificity, and G-Mean for the NEV and SEK groups.

Classification of skin lesions in the Malignant group. This group is divided into 3 subgroups: MEL and BCC + SCC group, BCC and MEL + SCC group, and SCC and MEL + BCC group, as shown in Table 7, with evaluation indicators: Accuracy, Precision, Recall, F1-score, Balanced Accuracy, Specificity, and G-Mean.

Classification of skin lesions in the Benign group. This group is divided into 3 subgroups: BCC and MEL + SCC; MEL and BCC + SCC; SCC and MEL + BCC. Of these 3 groups, the highest-performing group is the MEL and BCC + SCC group. In this group, the results are from BCC and SCC. Table 8 presents the performance of the 4 models, evaluated using the following indicators: Accuracy, Precision, Recall, F1-score, Balanced Accuracy, Specificity, and G-Mean for the BCC and SCC groups.

**Figure 5:**
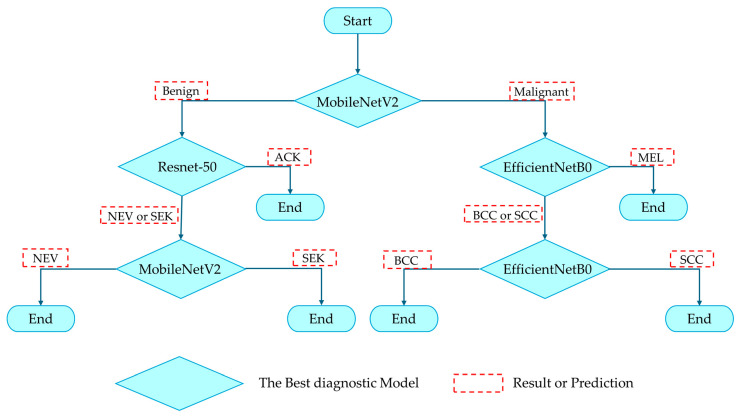
The flowchart illustrates the Hierarchical Classification Model, wherein the best model from each group is sequentially arranged to provide a classification strategy for the six types of dermatological lesions.

Based on the detailed performance evaluation of the models, the most effective model varied across hierarchical subgroups. Therefore, a hierarchical classification structure was designed by selecting the optimal model for each stage to maximize classification accuracy. At the first level, MobileNetV2, identified as a high-performing model, was employed to distinguish between the Benign and Malignant categories. For more complex subgroups, such as ACK and NEV + SEK, ResNet-50 was selected for its strong ability to learn deep, intricate features. The classification of NEV and SEK utilized MobileNetV2, while the more challenging classifications within the Malignant group, specifically MEL and BCC + SCC, were addressed using EfficientNetB0. Furthermore, EfficientNetB0 was also chosen for differentiating between BCC and SCC, given its balanced and effective scaling properties. The integrated system composed of these models is illustrated in Figure 5.

**Figure 6:**
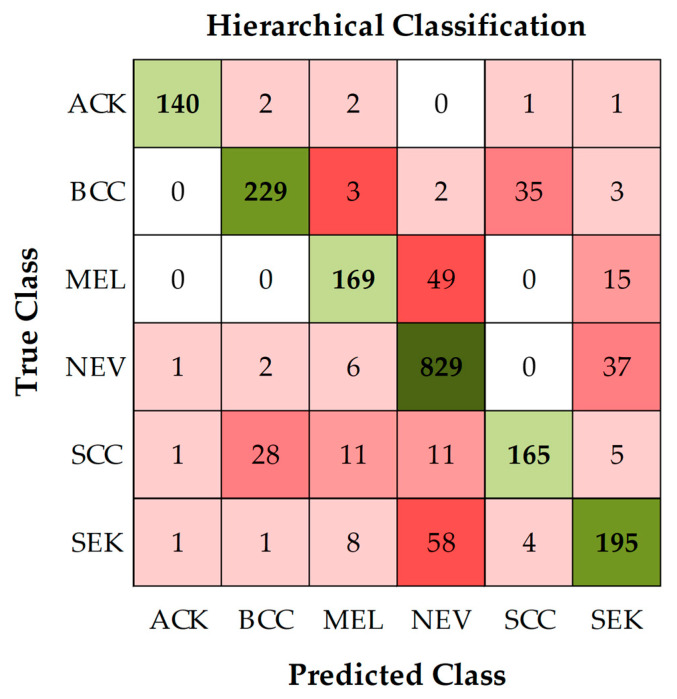
The confusion matrix displays the classification results of the hierarchical system between the Benign and Malignant lesion groups.

For performance evaluation, the confusion matrix in Figure 6 shows the system’s hierarchical classification results for each class. The results demonstrate that the system can effectively handle the complexity of dermatological disease classification.

The empirical comparison clearly highlights the superiority of the hierarchical approach over conventional multi-class classification methods. A multi-class classification model relies on a single model to distinguish all classes simultaneously, resulting in lower overall performance metrics due to the difficulty of learning diverse features simultaneously. In contrast, the hierarchical approach employs models optimized for each classification level, enabling more effective learning of the critical distinctions within each subgroup. Consequently, the models achieve significantly higher performance metrics, as shown in Table 9. These findings reinforce that deploying specialized models within a hierarchical structure is the most effective strategy for addressing this dermatological disease classification problem.

Additionally, as shown in Table 6, for the NEV vs. SEK classification task, MobileNetV2 achieved the highest recall; however, EfficientNetB0 outperformed it across the remaining evaluation metrics. An additional hierarchical strategy was evaluated at this classification node, with the results presented as Structure-2 in Table 9. Furthermore, Table 8 indicates that MobileNetV2 achieved significantly higher accuracy and precision, and it was therefore selected for further comparison as Structure-3, as shown in Table 9. Finally, both approaches were combined to establish Structure-4 in Table 9, which was compared with other strategies, including the selected baseline strategy (Structure-1), based on recall-driven selection criteria.

### 3.2. Grad-Cam

**Figure 7:**
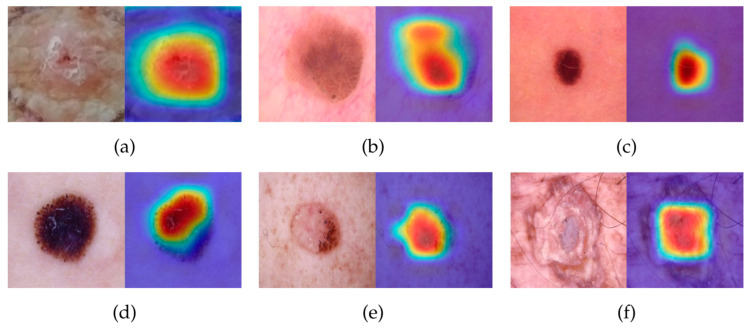
Grad-CAM visualization of skin disease: (**a**) Actinic Keratosis, (**b**) Nevus, (**c**) Seborrheic Keratosis, (**d**) Melanoma, (**e**) Basal Cell Carcinoma, and (**f**) Squamous Cell Carcinoma.

The classification of skin diseases using a hierarchical modeling approach, employing five trained and selected models, was confirmed to be accurate using Grad-CAM techniques. A radial color map is employed to highlight the regions that most influence the network’s decision-making process. Areas displayed in red and yellow indicate regions of peak activation where the network detected abnormalities, whereas blue areas represent minimal activation or normal tissue. As shown in Figure 7, the visualization provides a clear representation of six types of skin diseases, with the left image showing the lesion and the right showing the Grad-CAM visualization. These results serve to confirm and validate the accuracy of the network’s image analysis process.

## 4. Discussion

The results of this work highlight the effectiveness of hierarchical classification models in improving image classification performance across different skin diseases and skin cancer types. Splitting the classification task into a set of binary classification tasks, rather than relying on a multi-class model, significantly improved overall prediction accuracy and reliability.

As shown in the Supplementary Materials, the training results clearly demonstrate the effectiveness of the data preparation process. Although the augmented training dataset involves extensive transformations applied to the original dataset after splitting into training and testing sets, both training and testing results achieved comparable levels of accuracy. This indicates that the applied procedures do not introduce negative effects. Moreover, class imbalance can be effectively mitigated through these straightforward augmentation techniques.

In the results of the traditional single-model-based multi-class classification for identifying six types of dermatological images, the MobileNetV2 demonstrates the most reliable performance among the evaluated models. It achieved the highest accuracy, precision, F1-score, balanced accuracy, and G-mean, indicating optimal balanced performance on the classification task compared to other models. EfficientNetB0 performs moderately well but remains consistently below MobileNetV2, while both ResNet variants exhibit substantial performance degradation, particularly on metrics that reflect overall diagnostic reliability.

For multi-class classification, all investigated CNN models demonstrated relatively low performance on the skin disease dataset, with MobileNetV2 achieving the highest recall of 60.27%. This result suggests that the models struggled to handle the complexity of improving generalizability across image data from two different sources. Moreover, ambiguous features may arise when different classes share similar characteristics, further reducing classification performance. However, recall is a primary indicator of evaluation. Therefore, MobileNetV2 was selected for the performance evaluation of the proposed hierarchical classification model.

The results for the hierarchical classification model of the assembled structure of various binary classification models can be divided into five groups. Firstly, for classifying benign and malignant lesions, MobileNetV2 stands out as a remarkable model across multiple evaluation metrics, achieving scores that are closely aligned across all evaluations, with precision peaking at 92.70%, indicating robustness in predicting benign cases as incorrect. In addition, the model achieved the highest recall of 87.22%.

For the Benign group, the section with the highest performance is the ACK and NEV + SEK group, and the performance improved even further. The ResNet-50 model achieved the highest recall of 98.64%. These results imply that the model can most accurately distinguish ACK from other benign conditions. In addition, ResNet-50 achieved notably high scores in F1-score, Balanced Accuracy, and G-Mean. These metrics are key indicators of a classification model’s balanced performance. The F1-score, as the harmonic mean of precision and recall, reflects the model’s balance between prediction accuracy and coverage.

Meanwhile, balanced accuracy and G-mean are specifically designed to evaluate performance on imbalanced datasets by averaging Recall and Specificity. Together, these three metrics ensure that the model performs robustly not only on the majority class but also accurately identifies instances of the minority class. After successfully isolating ACK from the other benign conditions, it further classifies the remaining benign diseases, distinguishing between SEK and NEV. While EfficientNetB0 achieved the highest scores in most other metrics, MobileNetV2 delivered the highest Recall. Given that MobileNetV2’s performance across the different metrics was slightly lower than EfficientNetB0, it demonstrates that MobileNetV2 has overall effectiveness comparable to EfficientNetB0. Therefore, MobileNetV2 was selected for its superior recall of 93.06%.

Focus on malignant lesions, the group with the highest performance is the MEL and BCC + SCC group. EfficientNetB0 achieved the highest scores across all evaluation metrics, including the highest Recall. The EfficientNetB0 model achieved the highest Recall of 99.02%. These results imply that the model can most accurately distinguish MEL from other malignant conditions. After successfully isolating MEL from the other malignant conditions, it further classifies the remaining malignant cancers, distinguishing between BCC and SCC. As a result, the highest values are not found in a single model but lie between MobileNetV2 and EfficientNetB0. For evaluation, however, the most considered recall metric is achieved at 87.16% by EfficientNetB0. Furthermore, a higher mean F1-score and G-mean for this model indicate better balancing performance. For this reason, the model was chosen for further evaluation as an assembled architecture.

Therefore, the model was structured as a hierarchical classification system, utilizing the best-performing model for each subtask. This hierarchical binary classification achieved a recall of 82.62%, demonstrating superior performance over the multi-class approach by a significant margin of 22.35%. This layered framework improves interpretability and enables more advanced decision support, where early-stage prognoses can inform better-targeted assessments in later stages.

Sahithi et al. [49] proposed a hybrid convolutional and ensemble learning approach, achieving an accuracy of 92%. Mohamed et al. [50] incorporated metadata as additional information, enhancing the recall to up to 95%. Putri et al. [51] introduced a more sophisticated architecture that combines CNN and LSTM, attaining an accuracy of 97%.

While these hybrid approaches—leveraging metadata and increasingly complex network architectures—demonstrate improved performance, they often come at the cost of increased model complexity and reduced interpretability. In contrast, our proposed hierarchical-based models can be implemented in a straightforward manner without requiring modifications to the underlying neural network architectures. Since the individual networks are trained separately, this framework not only simplifies implementation but also provides clearer insights into the performance of each class pair, while achieving a substantial improvement in classification performance.

The Grad-CAM technique, as shown in Figure 7, supports quantitative results by demonstrating the transparency of the decision-making process. The red and yellow regions within the Grad-CAM maps clearly highlight the abnormal areas, confirming that the hierarchical classification accounts for clinical features relevant to the diagnosis. Consequently, this study concludes that the proposed hierarchical approach is both effective and reliable for computer-aided skin disease diagnosis and is particularly beneficial for classifying all six types of skin diseases.

Regardless of how decent the result shows, limitations remain. Exterior confirmation using clinical datasets from various residents’ involvement would be valuable for evaluating model generalizability in additional comprehensive settings. Eventually, this study’s findings show that hierarchical binary deep learning frameworks yield better results than conventional multi-class classification models for classifying skin diseases and cancer. As early detection can significantly improve patient outcomes in dermatology, the results encouraged further research and the clinical translation of AI-based diagnostic tools.

## 5. Conclusions

In conclusion, this work proposes novel methods for enhancing classification performance using a hierarchical model architecture, compared with conventional single-model approaches, for skin cancer and other relevant lesion image classification tasks. As shown by the results of the investigated models, a single model-based six-class image classification achieves moderate performance at a similar level. In contrast, a highly accurate potential can be achieved from almost binary classification models. Furthermore, the proposed assembled model significantly improves classification performance over traditional methods. Finally, this work may be beneficial for other challenging image classification tasks. Future studies could further investigate the limitations of the proposed models by increasing the scale and complexity of the problem, as well as evaluating additional inference-related factors, such as inference time, computational requirements, and expert validation for clinical implementation.

## Figures and Tables

**Table 1:** The model training hyperparameters.

Parameter	Value
GradientDecayFactor	0.9
SquaredGradientDecayFactor	0.999
Epsilon	1.00 × 10^−8^
InitialLearnRate	1.00 × 10^−3^
MaxEpochs	300
LearnRateSchedule	‘none’
LearnRateDropFactor	0.1
LearnRateDropPeriod	10
MiniBatchSize	40
Shuffle	‘every-epoch’
CheckpointFrequency	1
CheckpointFrequencyUnit	‘epoch’
SequenceLength	‘longest’
PreprocessingEnvironment	‘serial’
L2Regularization	1.00 × 10^−4^
GradientThresholdMethod	‘l2norm’
GradientThreshold	Inf
VerboseFrequency	50
ValidationFrequency	10
ValidationPatience	Inf
ObjectiveMetricName	‘loss’
SequencePaddingValue	0
SequencePaddingDirection	‘right’
InputDataFormats	“auto”
TargetDataFormats	“auto”
ResetInputNormalization	1
BatchNormalizationStatistics	‘auto’

**Table 2:** Specification Details of the Computing Devices Utilized for Model Training and Performance Evaluation.

Device	CPU	RAM	GPU
1	Intel Core i7-11800H (2.30 GHz)	16 GB	GeForce RTX 3050 Ti (4 GB)
2	Intel Core i5-13450HX (2.40 GHz)	32 GB	GeForce RTX 5050 (8 GB)
3	Intel Core i7-2600 (3.40 GHz)	24 GB	GeForce GTX 1650 (4 GB)

**Table 3:** Evaluation results of the CNN models for Multi-class Classification of skin lesions using key performance metrics.

Model	Accuracy	Precision	Recall	F1-Score	Specificity	Bal. Acc.	G-Mean
MobileNetV2	60.29%	68.50%	60.27%	62.63%	93.64%	76.95%	75.12%
EfficientNetB0	59.01%	67.29%	58.91%	60.86%	93.42%	76.16%	74.18%
ResNet-18	46.96%	54.45%	46.05%	48.50%	90.83%	68.44%	64.67%
ResNet-50	47.42%	56.87%	47.42%	49.29%	91.36%	69.39%	65.82%

**Table 4:** Evaluation results of the four CNN models for Binary Classification of skin lesions, comparing the Benign and Malignant groups using key performance metrics.

Model	Accuracy	Precision	Recall	F1-Score	Specificity	Bal. Acc.	G-Mean
MobileNetV2	84.30%	92.70%	87.22%	89.88%	85.43%	86.32%	86.32%
EfficientNetB0	74.42%	81.21%	81.65%	81.43%	66.98%	74.32%	73.96%
ResNet-18	68.43%	74.22%	79.34%	76.69%	58.96%	69.15%	68.39%
ResNet-50	69.96%	74.46%	77.84%	76.11%	57.93%	67.88%	67.15%

**Table 5:** Evaluation results of the four CNN models for binary classification of skin lesions, comparing the benign groups into 3 subgroups using key performance metrics.

Group	Model	Accuracy	Precision	Recall	F1-Score	Specificity	Bal. Acc.	G-Mean
ACK and NEV + SEK	MobileNetV2	99.87%	100.00%	97.99%	98.98%	100.00%	98.99%	98.99%
	EfficientNetB0	99.65%	100.00%	94.81%	97.33%	100.00%	97.40%	97.37%
	ResNet-18	99.05%	98.63%	96.00%	97.30%	99.82%	97.91%	97.89%
	ResNet-50	99.57%	99.32%	98.64%	98.98%	99.91%	99.28%	99.27%
NEV and ACK + SEK	MobileNetV2	86.95%	95.20%	90.45%	92.76%	88.56%	89.50%	89.50%
	EfficientNetB0	89.44%	90.74%	94.19%	92.43%	81.80%	87.99%	87.77%
	ResNet-18	81.93%	90.97%	87.67%	89.29%	79.21%	83.44%	83.33%
	ResNet-50	85.29%	93.83%	89.53%	91.63%	85.44%	87.49%	87.46%
SEK and ACK + NEV	MobileNetV2	85.85%	88.54%	95.26%	91.78%	65.49%	80.37%	78.98%
	EfficientNetB0	88.93%	94.71%	95.55%	95.13%	80.43%	87.99%	87.67%
	ResNet-18	81.08%	85.01%	93.43%	89.03%	57.38%	75.41%	73.22%
	ResNet-50	83.56%	84.72%	94.85%	89.50%	58.51%	76.68%	74.50%

**Table 6:** Evaluation results of the four CNN models for sub-classification within the Benign group, comparing the NEV and SEK group using key performance metrics.

Model	Accuracy	Precision	Recall	F1-Score	Specificity	Bal. Acc.	G-Mean
MobileNetV2	85.94%	95.09%	93.06%	94.06%	82.66%	87.86%	87.71%
EfficientNetB0	86.07%	96.11%	92.93%	94.49%	85.65%	89.29%	89.22%
ResNet-18	80.12%	89.83%	90.87%	90.34%	67.87%	79.37%	78.53%
ResNet-50	80.88%	93.60%	90.60%	92.07%	76.47%	83.53%	83.23%

**Table 7:** Evaluation results of the four CNN models for Binary Classification of skin lesions, comparing the Malignant groups into 3 subgroups using key performance metrics.

Group	Model	Accuracy	Precision	Recall	F1-Score	Specificity	Bal. Acc.	G-Mean
MEL and BCC + SCC	MobileNetV2	97.54%	71.83%	98.26%	82.99%	61.99%	80.13%	78.04%
	EfficientNetB0	98.83%	76.90%	99.02%	86.57%	66.79%	82.90%	81.32%
	ResNet-18	95.41%	75.13%	97.37%	84.81%	64.49%	80.93%	79.24%
	ResNet-50	96.45%	67.77%	98.16%	80.18%	58.77%	78.46%	75.95%
BCC and MEL + SCC	MobileNetV2	79.01%	62.87%	88.60%	73.55%	81.05%	84.83%	84.74%
	EfficientNetB0	82.54%	76.10%	80.54%	78.26%	86.14%	83.34%	83.30%
	ResNet-18	73.43%	60.29%	72.89%	66.00%	78.44%	75.67%	75.62%
	ResNet-50	76.60%	82.72%	62.67%	71.32%	87.19%	74.93%	73.92%
SCC and MEL + BCC	MobileNetV2	69.51%	86.53%	80.63%	83.48%	63.04%	71.84%	71.30%
	EfficientNetB0	73.46%	77.23%	85.34%	81.08%	57.25%	71.29%	69.90%
	ResNet-18	67.60%	76.83%	80.83%	78.78%	52.44%	66.64%	65.11%
	ResNet-50	62.26%	81.98%	76.52%	79.16%	50.81%	63.67%	62.36%

**Table 8:** Evaluation results of the four CNN models for sub-classification within the Malignant group, comparing the BCC and SCC groups using key performance metrics.

Model	Accuracy	Precision	Recall	F1-Score	Specificity	Bal. Acc.	G-Mean
MobileNetV2	85.31%	91.47%	82.23%	86.61%	87.85%	85.04%	84.99%
EfficientNetB0	81.21%	86.82%	87.16%	86.99%	83.89%	85.52%	85.51%
ResNet-18	80.00%	82.17%	62.35%	70.90%	64.06%	63.21%	63.20%
ResNet-50	67.35%	85.27%	72.61%	78.43%	76.97%	74.79%	74.76%

**Table 9:** Comparison of performance results between multi-class classification and hierarchical classification based on key performance metrics.

Classification Strategy		Model Performances						
		Accuracy	Precision	Recall	F1-Score	Specificity	Bal. Acc.	G-Mean
Multi–Class Classification		60.29%	68.50%	60.27%	62.63%	93.64%	76.96%	75.12%
Hierarchical Classification (Proposed model)	Structure-1	85.75%	85.71%	82.62%	83.97%	96.67%	89.65%	89.37%
	Structure-2	85.15%	85.21%	81.47%	83.02%	89.00%	96.53%	88.68%
	Structure-3	85.65%	85.66%	82.33%	83.85%	89.47%	96.62%	89.19%
	Structure-4	85.25%	85.20%	81.60%	83.09%	89.08%	96.57%	88.77%

## Data Availability

The original contributions presented in this study are included in the article, and further inquiries may be directed to the corresponding author.

## Supplementary Materials

The following supporting information can be downloaded at: https://www.mdpi.com/article/10.3390/s26092833/s1, Table S1. The results of the CNN models for Multi-class Classification. Table S2. The results of four CNN models for Binary Classification of skin lesions, comparing the Benign and Malignant groups. Table S3. The results of four CNN models for binary classification of skin lesions, comparing the benign groups into 3 subgroups. Table S4. The results of four CNN models for sub-classification within the Benign group, comparing the NEV and SEK group. Table S5. The results of four CNN models for Binary Classification of skin lesions, comparing the Malignant groups into 3 subgroups. Table S6. The results of four CNN models for sub-classification within the Malignant group, comparing the BCC and SCC group.
