# Supplementary material for: Hierarchical Deep Learning Framework for Skin Disease and Cancer Classification Performance Enhancement
Source: Sensors (Basel). 2026 May 1;26(9):2833. doi: 10.3390/s26092833 (PMC13165980; doi:10.3390/s26092833)
Supplement: Supplementary file 1 [file sensors-26-02833-s001.zip › sensors-4256414-supplementary.pdf]

1. Multi-class Classification

Table S1. The results of the CNN models for Multi-class Classification. (6 Classes)

Model	Accuracy		Precision	Recall	F1-score	Specificity	Bal. Acc.	G-Mean
	Train	Test						
MobileNetV2	57.81%	60.29%	68.50%	60.27%	62.63%	93.64%	76.95%	75.12%
EfficientNetB0	57.24%	59.01%	67.29%	58.91%	60.86%	93.42%	76.16%	74.18%
ResNet-18	49.62%	46.96%	54.45%	46.05%	48.50%	90.83%	68.44%	64.67%
ResNet-50	64.71%	47.42%	56.87%	47.42%	49.29%	91.36%	69.39%	65.82%

2. Hierarchical Binary Classification

Table S2. The results of four CNN models for Binary Classification of skin lesions, comparing the Benign and Malignant groups. (BM)

Model	Accuracy		Precision	Recall	F1-score	Specificity	Bal. Acc.	G-Mean
	Train	Test						
MobileNetV2	84.57%	84.30%	92.70%	87.22%	89.88%	85.43%	86.32%	86.32%
EfficientNetB0	78.29%	74.42%	81.21%	81.65%	81.43%	66.98%	74.32%	73.96%
ResNet-18	67.75%	68.43%	74.22%	79.34%	76.69%	58.96%	69.15%	68.39%
ResNet-50	70.68%	69.96%	74.46%	77.84%	76.11%	57.93%	67.88%	67.15%

Table S3. The results of four CNN models for binary classification of skin lesions, comparing the benign groups into 3 subgroups.

Group	Model	Accuracy		Precision	Recall	F1-score	Specificity	Bal. Acc.	G-Mean
		Train	Test						
	MobileNetV2	99.86%	99.87%	100.00%	97.99%	98.98%	100.00%	98.99%	98.99%
ACK and NEV +	EfficientNetB0	99.14%	99.65%	100.00%	94.81%	97.33%	100.00%	97.40%	97.37%
SEK	ResNet-18	98.57%	99.05%	98.63%	96.00%	97.30%	99.82%	97.91%	97.89%
	ResNet-50	99.71%	99.57%	99.32%	98.64%	98.98%	99.91%	99.28%	99.27%
	MobileNetV2	89.57%	86.95%	95.20%	90.45%	92.76%	88.56%	89.50%	89.50%
NEV and	EfficientNetB0	89.57%	89.44%	90.74%	94.19%	92.43%	81.80%	87.99%	87.77%
ACK+SEK	ResNet-18	82.00%	81.93%	90.97%	87.67%	89.29%	79.21%	83.44%	83.33%
	ResNet-50	81.29%	85.29%	93.83%	89.53%	91.63%	85.44%	87.49%	87.46%
	MobileNetV2	88.29%	85.85%	88.54%	95.26%	91.78%	65.49%	80.37%	78.98%
SEK and	EfficientNetB0	90.71%	88.93%	94.71%	95.55%	95.13%	80.43%	87.99%	87.67%
ACK+NEV	ResNet-18	83.57%	81.08%	85.01%	93.43%	89.03%	57.38%	75.41%	73.22%
	ResNet-50	87.86%	83.56%	84.72%	94.85%	89.50%	58.51%	76.68%	74.50%

Table S4. The results of four CNN models for sub-classification within the Benign group, comparing the NEV and SEK group.

Model	Accuracy		Precision	Recall	F1-score	Specificity	Bal. Acc.	G-Mean
	Train	Test						
MobileNetV2	89.71%	85.94 %	95.09%	93.06%	94.06%	82.66%	87.86%	87.71%
EfficientNetB0	87.29%	86.07 %	96.11%	92.93%	94.49%	85.65%	89.29%	89.22%
ResNet-18	85.57%	80.12 %	89.83%	90.87%	90.34%	67.87%	79.37%	78.53%
ResNet-50	85.86%	80.88 %	93.60%	90.60%	92.07%	76.47%	83.53%	83.23%

Table S5. The results of four CNN models for Binary Classification of skin lesions, comparing the Malignant groups into 3 subgroups.

Group	Model	Accuracy		Precision	Recall	F1-score	Specificity	Bal. Acc.	G-Mean
		Train	Test						
MEL and BCC + SCC	MobileNetV2	95.00%	97.54 %	71.83%	98.26%	82.99%	61.99%	80.13%	78.04%
	EfficientNetB0	94.71%	98.83%	76.90%	99.02%	86.57%	66.79%	82.90%	81.32%
	ResNet-18	90.43%	95.41%	75.13%	97.37%	84.81%	64.49%	80.93%	79.24%
	ResNet-50	90.86%	96.45%	67.77%	98.16%	80.18%	58.77%	78.46%	75.95%
BCC and MEL+SCC	MobileNetV2	74.71%	79.01%	62.87%	88.60%	73.55%	81.05%	84.83%	84.74%
	EfficientNetB0	73.00%	82.54%	76.10%	80.54%	78.26%	86.14%	83.34%	83.30%
	ResNet-18	64.00%	73.43%	60.29%	72.89%	66.00%	78.44%	75.67%	75.62%
	ResNet-50	75.57%	76.60%	82.72%	62.67%	71.32%	87.19%	74.93%	73.92%
SCC and MEL+BCC	MobileNetV2	86.14%	69.51%	86.53%	80.63%	83.48%	63.04%	71.84%	71.30%
	EfficientNetB0	73.46%	73.46%	77.23%	85.34%	81.08%	57.25%	71.29%	69.90%
	ResNet-18	67.60%	67.60%	76.83%	80.83%	78.78%	52.44%	66.64%	65.11%
	ResNet-50	62.26%	62.26%	81.98%	76.52%	79.16%	50.81%	63.67%	62.36%

Table S6. The results of four CNN models for sub-classification within the Malignant group, comparing the BCC and SCC group

Model	Accuracy		Precision	Recall	F1-score	Specificity	Bal. Acc.	G-Mean
	Train	Test						
MobileNetV2	89.14%	85.31 %	91.47%	82.23%	86.61%	87.85%	85.04%	84.99%
EfficientNetB0	86.71%	81.21 %	86.82%	87.16%	86.99%	83.89%	85.52%	85.51%
ResNet-18	82.00%	80.00 %	82.17%	62.35%	70.90%	64.06%	63.21%	63.20%
ResNet-50	82.57%	67.35 %	85.27%	72.61%	78.43%	76.97%	74.79%	74.76%
